# Supplementary material for: High sensitivity of ultrasound for the diagnosis of tuberculosis in adults in South Africa: A proof-of-concept study
Source: PLOS Glob Public Health. 2022 Oct 6;2(10):e0000800. doi: 10.1371/journal.pgph.0000800 (PMC10021214; doi:10.1371/journal.pgph.0000800)
Supplement: S2 Table — (DOCX) [file pgph.0000800.s002.docx]

S2 Table: Ultrasound findings in participants with bacteriologic TB compared to healthy participants (n = 71)

| **Ultrasound finding - no. (%)** |  |  | | **Bacteriologic TB** |  |
| --- | --- | --- | --- | --- | --- |
|  | **TB** | **Healthy** | | ***Sensitivity***  ***(95% CI^*^)*** | ***Specificity***  ***(95% CI)*** |
|  | ***(N=34)*** | ***(N=37)*** | |  |  |
| 1. **A. Composite Scores:** |  |  | |  |  |
| **Any Thoracic or FASH**^†^ **Pathology**^^^ | 31 (91) | 17 (46) | | 0.91 (0.76–0.98) | 0.54 (0.37–0.71) |
| **Any Thoracic Pathology**^^^ | 29 (85) | | 14 (38) | 0.85 (0.69–0.95) | 0.62 (0.45–0.78) |
| **Thoracic Combo 1**^^^ | 26 (76) | 6 (16) | | 0.76 (0.59–0.89) | 0.84 (0.68–0.94) |
| **Thoracic Combo 2**^^^ | 23 (68) | 6 (16) | | 0.68 (0.49–0.83) | 0.84 (0.68–0.94) |
| **Thoracic Combo 3**^^^ | 25 (74) | 1 (3) | | 0.74 (0.56–0.87) | 0.97 (0.86–1) |
| **FASH Combo 1**^^^ | 7 (21) | 4 (11) | | 0.21 (0.09–0.38) | 0.89 (0.75–0.97) |
| **Thoracic Combo 1 or FASH Combo 1**^^^ | 27 (79) | 10 (27) | | 0.79 (0.62–0.91) | 0.73 (0.56–0.86) |
| **Any FASH Pathology**^^^ | 8 (24) | 4 (11) | | 0.24 (0.11–0.41) | 0.89 (0.75–0.97) |
| **B. Specific Findings:** |  |  | |  |  |
| **Consolidation** | 22 (65) | 0 (0) | | 0.65 (0.46–0.8) | 1 (0.91–1) |
| **Consolidation, Upper** | 13 (38) | 0 (0) | | 0.38 (0.22–0.56) | 1 (0.91–1) |
| **Small Subpleural Consolidation (SPC)** | 19 (56) | 6 (16) | | 0.56 (0.38–0.73) | 0.84 (0.68–0.94) |
| **SPC, Upper** | 9 (26) | 1 (3) | | 0.26 (0.13–0.44) | 0.97 (0.86–1) |
| **SPC, 2 or more** | 13 (38) | 1 (3) | | 0.38 (0.22–0.56) | 0.97 (0.86–1) |
| **B1 or B2 pattern** | 5 (15) | 3 (8) | | 0.15 (0.05–0.31) | 0.92 (0.78–0.98) |
| **B2 pattern** | 0 (0) | 0 (0) | | 0 (0–0.1) | 1 (0.91–1) |
| **Irregular Pleural Line** | 22 (65) | 9 (24) | | 0.65 (0.46–0.8) | 0.76 (0.59–0.88) |
| **Pleural Effusion** | 4 (12) | 0 (0) | | 0.12 (0.03–0.27) | 1 (0.91–1) |
| **Cavity** | 3 (9) | 0 (0) | | 0.09 (0.02–0.24) | 1 (0.91–1) |
| **Pericardial Effusion** | 6 (18) | 4 (11) | | 0.18 (0.07–0.35) | 0.89 (0.75–0.97) |
| **Hepatic Lesions** | 1 (0.3) | 0 (0) | | 0.03 (0–0.15) | 1 (0.91–1) |
| **Periaortic Lymphadenopathy** | 1 (0.3) | 0 (0) | | 0.03 (0–0.15) | 1 (0.91–1) |
| **Ascites** | 2 (0.6) | 0 (0) | | 0.06 (0.01–0.2) | 1 (0.91–1) |

* CI = confidence interval

† FASH = Focused Assessment with Sonography for HIV-Associated Tuberculosis

^ Refer to Table 1 for definitions of composite scores
