# Supplementary material for: High sensitivity of ultrasound for the diagnosis of tuberculosis in adults in South Africa: A proof-of-concept study
Source: PLOS Glob Public Health. 2022 Oct 6;2(10):e0000800. doi: 10.1371/journal.pgph.0000800 (PMC10021214; doi:10.1371/journal.pgph.0000800)
Supplement: Attachment — Submitted filename: Response to Editor Comments June.17.2022.docx [file pgph.0000800.s004.docx]

PGPH-D-22-00300R1

High sensitivity of ultrasound for diagnosis of tuberculosis in adults in South Africa: a proof-of-concept study

PLOS Global Public Health

Response to Editor and Reviewer’s Comments

RESPONSE TO EDITOR

Dear Dr. Kherkoff,

Thank you very much for the review of our manuscript.

I have incorporated changes into the manuscript using track changes, as requested, to indicate those portions of the text that have been revised.

Please find attached a point-by-point response to your comments. Your original comments are in italics, and our responses are in regular font.

Sincerely,

Matthew Fentress

RESPONSE TO EDITOR’S COMMENTS

We greatly appreciate your helpful suggestions to improve our manuscript. Our responses to your comments are below.

*I thank the authors for their resubmission and thoughtful revisions. I have reviewed the revised manuscript and I believe it has been substantially strengthened and can be accepted for publication pending addressing the minor points outlined below. Because there is no formal editing service during the publication process, I kindly ask the authors to carefully review their manuscript one final time for any grammar or typographical errors.


Title:
- Add “the” to title: High sensitivity of ultrasound for the diagnosis of tuberculosis in adults in South Africa.
- It would also be informative to make clear that this is a pilot/proof-of-concept study. Please consider adding a descriptive study title after the primary study title, (alternative wording also ok) e.g., "High sensitivity of ultrasound for the diagnosis of tuberculosis in adults in South Africa: a proof-of-concept study"*

**Response 1:** We have amended the title to read as follows: “High sensitivity of ultrasound for the diagnosis of tuberculosis in adults in South Africa: a proof-of-concept study”

*Abstract:
- Please break this sentence into two sentences or add semi colon: e.g., “We performed comprehensive thoracic and focused abdominal ultrasound examinations were performed by trained radiologists and pulmonologists on adults recruited from a community multimorbidity survey and a primary healthcare clinic in KwaZulu-Natal Province, South Africa. Sputum samples were systematically collected from all participants.”*

**Response 2:** We have broken this into two sentences, as follows**: “**Comprehensive thoracic and focused abdominal ultrasound examinations were performed by trained radiologists and pulmonologists on adults recruited from a community multimorbidity survey and a primary healthcare clinic in KwaZulu-Natal Province, South Africa. Sputum samples were systematically collected from all participants.”

*- Please consider acknowledging the setting (e.g., South Africa) or that it is a high burden setting, e.g., “A comprehensive ultrasound scanning protocol in adults in a high TB burden setting had high sensitivity but low specificity to identify bacteriologically-confirmed tuberculosis.”*

**Response 3:** We have acknowledged the setting, and the sentence now reads: “A comprehensive ultrasound scanning protocol in adults in a high TB burden setting had high sensitivity but low specificity to identify bacteriologically-confirmed tuberculosis.”

*Author summary:
- First sentence: Please acknowledge that these limitations in diagnostic access are often at lower health care levels where patients may first present and clarify what is meant by diagnosis is meant by where TB diagnosis is routinely performed. Are they suggesting a high level of clinical diagnoses/empiric treatment due to lack of available diagnostic tools?*

**Response 4:** We have adapted the sentence to address these concerns, and it now reads: “Standard tests for tuberculosis include sputum testing and chest radiography, but these tests are rarely available at the point of care in primary healthcare facilities where patients may first present, and where tuberculosis diagnosis is frequently performed in tuberculosis-prevalent settings.”

*- For the following sentence, please slightly reframe language for improved clarity, as high sensitivity suggests a role as a triage tool to help rule-in TB. “The high sensitivity of ultrasound for tuberculosis in this study suggests a potential for ultrasound to be used for diagnosis of tuberculosis, particularly in areas where chest radiography is not accessible.”*

**Response 5:** We have clarified the potential for use as a triage test, and the sentence now reads as follows: “The high sensitivity of ultrasound for tuberculosis in this study suggests a potential for ultrasound to be used as a triage test for tuberculosis, particularly in areas where chest radiography is not accessible.”

*Manuscript body:
- Please slightly revise the following sentence to remove the word major, “A 2021 systematic review of thoracic ultrasound for TB diagnosis found only six, mostly hospital-based studies that had methodological limitations, including small sample sizes and a lack of well characterized comparison groups.”*

**Response 6:** The word “major” has been removed.

*- Please slightly reframe this sentence to be consistent with a triage use case (rather than diagnosis), “If ultrasound has
appropriate performance characteristics for diagnosis of PTB, it could be incorporated into point-of-care algorithms to accelerate diagnosis and treatment of TB.”*

**Response 7:** The sentence has been reframed to be consistent with a triage use, and now reads as follows: “If ultrasound has appropriate performance characteristics for diagnosis of PTB, it could be incorporated into point-of-care algorithms for triage of TB, and potentially shorten the time to treatment initiation.”

*- Please add a comma after which. “Survey participants who reported any TB symptom (cough, weight loss, night sweats, or fever) or had a CAD4TB score above 25 were asked to give a sputum specimen, which was divided into two in the laboratory”*

**Response 8:** We have added a comma added before “which”. *-Table 1. Please reformat in a way that it is one single table (e.g., not two under a single Table 1 header)*

**Response 9:** The table has been reformatted so that it is a single table. In addition, a few minor formatting edits have been made to improve readability.

*- Please add a period to the end of the following sentence. “If radiological signs of previous TB were present, lower certainty was assigned to the classification of active TB”*

**Response 10:** A period has been added to the end of the sentence.

*- Under the “ultrasound findings” subsection, for consistency, I would report the findings pertaining to FASH/abdominal US in the same way you have for Thoracic US.*

**Response 11:** We added reporting of pericardial effusion results at the end of ultrasound findings subsection, for consistency with the reporting for thoracic ultrasound. Other individual FASH findings were not included in the text since the number of cases with pathology was very low (only 1 abdominal lymphadenopathy, 1 hepatic lesion, 2 ascites, 0 splenic lesions).

*-Table 2. Please move the total/overall column to left side before the active TB participants column.*

**Response 12:** The total column has been moved to the left side of the table.

In addition, a few minor formatting edits have been made to improve readability.

*-Table 3. Is there any way to restructure this table so that there is clearer hierarchy/is easier to follow? For example could you group any thoracic pathology directly preceded or followed by the individual features and similarly for any FASH pathology? The table is difficult to follow as is. Additionally, I think it would also be helpful to either include definitions of different combination findings directly in the footnote or reference that they can be found in table 1.
-Table 4. Similar point to Table 3. Are there opportunities for restructuring the table in a way that may make nested findings clearer? Also, while I think this is useful/important data to include, I believe that this may be more appropriate as a Supplementary Table, especially as it may be slightly confusing to some reviewers as to how this differs from Table 3.*

**Response 13:** We have changed the structure of tables 3 and 4 to make the hierarchy easier to follow. We have labeled the first group as “Composite Scores”, followed by “Specific Findings”. We have added hierarchical indentation for each group to improve clarity. Finally, we have added a footnote that references Table 1 for definitions of all composite scores. Regarding Table 4, we agree that this may be more appropriate as a supplement, therefore it has been moved to the Supplementary Data section, and it has been relabeled as Supplementary Table 2. In addition, a few minor formatting edits have been made to to these tables to improve readability.

*-The following sentence seems like it should be referenced. Are the relevant references the same as the following sentence? “Overall, our findings that thoracic ultrasound has high sensitivity and low specificity for TB are similar to results reported in prior studies.”*

**Response 14:** Yes, the relevant references are the same as the following sentences, plus a systematic review. We have added the relevant references to the end of this sentence.

*-For the following sentence, consider adding a very brief mention of how cavities are also associated with a high degree of infectiousness. “This represents an important limitation of ultrasound since cavity is a key radiological finding in pulmonary TB.”*

- **Response 15:** We have added language to mention the high degree of infectiousness, along with a reference (manuscript reference #24, PMID 32482293) regarding increased transmission risk with cavitary disease. The sentence now reads: “This represents an important limitation of ultrasound since cavity is a key radiological finding in pulmonary TB and increases the risk of transmission.”

*- As this is the discussion section, please remove table reference from the following sentence, “Overall performance did not appear to differ markedly between participants with and without HIV (Supplementary Table 1), although this subgroup analysis must be interpreted with caution given the small number of participants.*

**Response 16:** The table reference has been removed. *- Please don’t capitalize Voluntary Counseling and Testing in the following sentence “…in screening for extrapulmonary TB in those newly diagnosed with HIV through outpatient Voluntary Counseling and Testing (VCT).”*

**Response 17:** The capitalization has been removed.

In addition to the changes described above in direct response to the editor’s comments, we have made several grammar and syntax edits using track changes to the manuscript to improve readability.
