# Supplementary material for: High sensitivity of ultrasound for the diagnosis of tuberculosis in adults in South Africa: A proof-of-concept study
Source: PLOS Glob Public Health. 2022 Oct 6;2(10):e0000800. doi: 10.1371/journal.pgph.0000800 (PMC10021214; doi:10.1371/journal.pgph.0000800)
Supplement: Attachment — Submitted filename: PGPH-D-22-00300 - Response to Editor and Reviewer Comments .docx [file pgph.0000800.s003.docx]

PGPH-D-22-00300

High sensitivity of ultrasound for diagnosis of tuberculosis in adults in South Africa

PLoS Global Public Health

Response to Editor and Reviewer’s Comments

RESPONSE TO EDITOR

Dear Dr. Andrew Kherkoff,

Thank you very much for the review of our manuscript.

I have incorporated changes into the manuscript using track changes, as requested, to indicate those portions of the text that have been revised.

Please find attached a point-by-point response to the reviewer’s comments. The reviewer’s original comments are in italics, and our responses are in regular font.

Sincerely,

Matthew Fentress

RESPONSE TO EDITOR’S COMMENTS

We greatly appreciate your helpful suggestions to improve our manuscript. Our responses to your comments are below.

*Journal Requirements:
1. Please amend your detailed Financial Disclosure statement. This is published with the article, therefore should be completed in full sentences and contain the exact wording you wish to be published.*

*ii). State the initials, alongside each funding source, of each author to receive each grant.
iii). State what role the funders took in the study. If the funders had no role in your study, please state: “The funders had no role in study design, data collection and analysis, decision to publish, or preparation of the manuscript.”*

**Response 1:** We have amended the Financial Disclosure statement accordingly. The text now reads as follows (Page 3):

This work was supported, in whole or in part, by the Bill & Melinda Gates Foundation [Grant Number OPP1212544]. A.G. and E.W. are the PI and co-PI, respectively, for this grant. The funders were involved in the design of the study, but had no role in data collection, data analysis, decision to publish or preparation of the manuscript. Under the grant conditions of the Foundation, a Creative Commons Attribution 4.0 Generic License has already been assigned to the Author Accepted Manuscript version that might arise from this submission.

*Additional Editor Comments (if provided):

Abstract
- Please add sub-headers to provide improve structure*

**Response 2:** Sub-headers have been added.

*- First sentence: please clarify if you mean all forms of TB (PTB, EPTB, or both) and among all persons, or restricted to HIV-positive persons only.*

**Response 3:** This sentence has been clarified, and now reads as follows (Page 5):

There are limited data on the performance characteristics of ultrasound for the diagnosis of pulmonary tuberculosis in both HIV-positive and HIV-negative persons.

*- Please add slightly more details about the reference standard. Was it sputum-based only? Were samples systematically collected? Please also briefly mentioned who performed the US exams (e.g., was this highly skilled/experienced radiologists?)*

**Response 4:** We have clarified this sentence, and it now reads as follows (Page 5):

Comprehensive thoracic and focused abdominal ultrasound examinations were performed by trained radiologists and pulmonologists on adults recruited from a community multimorbidity survey and a primary healthcare clinic in KwaZulu-Natal Province, South Africa, and sputum samples were systematically collected from all participants.

*- I appreciate that numbers are small, but it would be appropriate add brief details to results as whether performance appeared to differ according to HIV status.*

**Response 5:** We have added this information to the manuscript, and included statements about this information in the Abstract, as well as in the Results and Discussion sections, and provided a Supplementary Table 1. These statements now read as follows:

Abstract (Page 5): Overall performance did not appear to differ markedly between participants with and without HIV.

Results (Page 18): Amongst HIV-positive participants, sensitivity and specificity of any thoracic or FASH ultrasound abnormality for TB was 90% (95% CI 68%-99%) and 32% (95% CI 14%-55%), while amongst HIV-negative participants it was 93% (95% CI 66-100%) and 57% (37%-76%) (Supplementary Table 1).

Discussion (Page 22): Overall performance did not appear to differ markedly between participants with and without HIV (Supplementary Table 1), although this subgroup analysis must be interpreted with caution given the small number of participants.

*Results
- Please clarify how many participants were enrolled from the community survey compared to those from the TB facility. This has important implications on pre-test probability of TB disease and how advanced TB disease may be.*

**Response 6:** This data has been added to the Results section, which now reads as follows (Page 16):

Of those enrolled, 61 participants were from the community survey (3 bacteriologic TB, 8 radiologic TB, 50 no TB), and 31 participants were from the primary care clinic (31 bacteriologic TB).

*- I know the number of participants was small, but did you evaluate the overall sens/spec by HIV status? I think if even as a supplementary table, this is important data to report. However, I do appreciate that there is not sufficient statistical power to draw any firm conclusions from such sub-group analyses.*

**Response 7:** This has been addressed as detailed in Response 5 above.

*Discussion:
- Please add additional discussion regarding challenges of implementing US as a screening tool for TB disease. E.g., it took on average 41 minutes for highly skilled radiologists/pulmonologists. Further how available is US compared to CXR at lower health levels in high burden settings?*

**Response 8:** We have added additional comment on the noted challenges, and referenced our group’s recently published article that explores these challenges in further detail. The paragraph now reads as follows (Page 24):

The potential logistical advantages of ultrasound over chest radiograph for diagnosis of TB include its portability, safety profile, low cost, need for minimal consumables, and ability to function on battery power in remote settings.^1^ CXR may not be available at lower levels of the health system in many high burden settings, and the portability and low cost of ultrasound could present a particular advantage in these settings. However, despite the potential advantages of ultrasound, there are a number of challenges to implementation of ultrasound as a diagnostic tool for TB disease, including the need for robust operator training, and the length of time required for the comprehensive scan technique used in this study.^2^

*- Somewhere is the discussion it is important to note that accuracy may be overestimated compared to real-world settings, given 1) they were not read under real-world clinical demands/settings and 2) they were by highly experienced study staff and not clinicians or clinical officers at the study facilities.*  *- Under limitations, please also discuss the lack of systematic testing for EPTB to define the reference standard. This is especially important to mention given you are evaluating the diagnostic accuracy, at least in part, of abdominal ultrasound. This may have resulted in the misclassification of patients.*

**Response 9:** The section on limitations has been adapted accordingly, and now reads as follows (Page 25):

The primary limitation of this study is a small sample size. The study was originally designed to recruit 200 participants, but we were forced to suspend recruitment in March 2020 due to the COVID-19 pandemic. In addition, since the study was designed to focus on ambulatory patients, the results may not be generalizable to a hospitalized population. The lack of systematic testing for extrapulmonary TB could have resulted in misclassification. Finally, the ultrasounds were performed and interpreted under idealized rather than real-world conditions, which may have resulted in an overestimation of diagnostic accuracy, although this was in keeping with the proof-of-concept design.

*- You mention AI for US on two separate occasions in your discussion. On the first mention, are there any references that be provided that show initial promise for this approach?*

**Response 10:** A reference has been added here (Page 24, manuscript reference #30) that reflects the promise of AI in lung ultrasound.^3^

*- You also note use of US as a screening tool on multiple occasions. However, it appears the authors are referring to use as a triage tool (e.g., in persons who are actively seeking care and who are largely symptomatic). It’s a bit difficult to tease out as they note enrolment of participants they may represent a screening use case (survey participants) use and others that represent a triage use (health-facility participants). Please clarify.*

**Response 11:** Thank you for pointing out this apparent discrepancy so that we could improve the clarity of the manuscript. We intended to evaluate the use of ultrasound primarily as a triage tool, rather than a screening tool. We have now adapted the language to clarify this throughout, and removed the word “screening” where it was not appropriate. The main reason for recruiting people from the community survey was because it was a good way to identify people who were definitely free of TB (i.e. negative sputum and CXR) in a population where subclinical TB is common, rather than because we were evaluating ultrasound as a screening test.

RESPONSE TO REVIEWER #1

We greatly appreciate your helpful suggestions to improve our manuscript. Our responses to your comments are below.

*Reviewer #1: I read with interest this paper. Authors wrote an interesting and well presented paper. The role of ultrasound in Tb diagnosis is an important topic. Thanks for treat this topic. Below my suggestions
1. Introduction: updata data on Tb following tb report 2021 and introduce better why the ultrasound can be useful in tb diagnosis.*

**Response 12:** The reference has been updated to the 2021 TB Report.

*2. Methods and results: excellent, clear , statistical analysis is perfect. Well done
3. Discussion: add the potential role of ultrasound also in abdominal tb in HIV patients as showed in a paper from South Sudan, (see and cite Bobbio F, Di Gennaro F, Marotta C, Kok J, Akec G, Norbis L, Monno L, Saracino A, Mazzucco W, Lunardi M. Focused ultrasound to diagnose HIV-associated tuberculosis (FASH) in the extremely resource-limited setting of South Sudan: a cross-sectional study. BMJ Open. 2019 Apr 2;9(4):e027179. doi: 10.1136/bmjopen-2018-027179. ).*

**Response 13:** We have added into the Discussion the potential role of TB in HIV patients, and included the Bobbio reference. This paragraph now reads as follows (Page 22):

Few of our study participants had abnormal abdominal ultrasound findings, likely related to a relatively low burden of extrapulmonary TB in this ambulatory population that included HIV-negative people. Despite these low numbers and wide confidence intervals, the trend of high specificity observed for abdominal findings is similar to studies which have used ultrasound to assess for HIV-associated TB in the emergency department or inpatient setting. ^4–6^ In addition to its utility in the acute care setting, ultrasound has also been suggested to have a potential role in screening for extrapulmonary TB in those newly diagnosed with HIV through outpatient Voluntary Counseling and Testing (VCT).^7^

*Furthermore, give same global health proposal that came from your very very good paper.*

**Response 14:** We have added language to further reflect the potential advantages of ultrasound in TB endemic settings, and included reference to an article which discusses these factors further. This paragraph now reads as follows (Page 24):

The potential logistical advantages of ultrasound over chest radiograph for diagnosis of TB include its portability, safety profile, low cost, need for minimal consumables, and ability to function on battery power in remote settings.^1^ CXR may not be available at lower levels of the health system in many high burden settings, and the portability and low cost of ultrasound could present a particular advantage in these settings. However, despite the potential advantages of ultrasound, there are a number of challenges to implementation of ultrasound as a diagnostic tool for TB disease, including the need for robust operator training, and the length of time required for the comprehensive scan technique used in this study.^2^

RESPONSE TO REVIEWER #2

We greatly appreciate your helpful suggestions to improve our manuscript. Our responses to your comments are below.

*Reviewer #2: - A systematically selected convenience sample was used which are likely to skew the results t`owards a higher sensitivity. A group of patients already diagnosed with TB were part of the included group who will further skew the results as no negative TB cases are possible (hence limiting the specificity and the use as a screening tool). Please include how many of these patients were included in the final sample - if the majority of the TB positive patients are from this group, this study is unfortunately nothing more that a case series.*

**Response 15:** The data regarding the number of participants recruited from each site has been added to the Results section, and reads as follows (Page 16):

Of those enrolled, 61 participants were from the community survey (3 bacteriologic TB, 8 radiologic TB, 50 no TB), and 31 participants were from the primary care clinic (31 bacteriologic TB).

*- Participants with suspected drug-resistant TB were excluded. Please include the definition of suspected drug-resistant TB and justify why they were excluded.*

**Response 16:** The paragraph on exclusion criteria has been adapted to include the definition of drug-resistant TB and the rationale for exclusion, and now reads as follows (Pages 9-10):

Exclusion criteria for all participants included known or suspected drug-resistant TB, as defined by rifampin resistance on Xpert MTB/RIF results; too unwell to undergo study procedures; and known or suspected pregnancy. The decision to exclude drug-resistant TB participants was based on operational considerations. Enrolled participants had prolonged close contact with study staff shortly after initiation of treatment. Since there is lower certainty that patients with drug-resistant TB will become noninfectious rapidly after treatment initiation compared to drug-sensitive TB, participants with drug-resistant TB were excluded to reduce the risk of TB transmission.

*- Beside the small sample which resulted in an under powered study, there are two more limitations to the study that need to be acknowledged. Ultrasounds were performed by radiologists or pulmonologists which limits the generalizability of the study, specifically as TB are very prevalent in resource-limited settings where these expertise might not be available nor affordable.*

**Response 17:** This point was also noted in the editor’s comments, and has been incorporated added to the limitations as follows (page 24):

Finally, the ultrasounds were performed and interpreted under idealized rather than real-world conditions, which may have resulted in an overestimation of diagnostic accuracy, although this was in keeping with the proof-of-concept design.

*Secondly, the thoracic ultrasound took 32 minutes (and the abdomen a further 7 minutes), this is rather long to implement as a screening tool.*

**Response 18:** This point was also noted in the editor’s comments (see Response 8), and language was added to acknowledge this challenge in the Discussion which reads as follows (page 23):

However, despite the potential advantages of ultrasound, there are a number of challenges to implementation of ultrasound as a diagnostic tool for TB disease, including the need for robust operator training, and the length of time required for the comprehensive scan technique used in this study.^2^

*Minor comments:
- The term reference standard is preferred over gold standard.*

**Response 19:** All occurrences of “gold” standard have been changed to “reference” standard.

*- Please be consistent in the use of thoracic abnormalities versus pulmonary abnormalities.*

**Response 20:** We have changed this to consistently use thoracic abnormalities throughout.

*- It is no surprise that the FASH scan performed poorly as half the sample were HIV-negative. The value of ultrasound as a screening test in ambulatory symptomatic people would be limited to pulmonary TB (i.e.thoracic ultrasound).
- The tables are rather long and the authors should consider to put ultrasound findings with limited numbers as supplementary material.*

**Response 21:** We appreciate the comment. However, for the sake of consistency, we prefer to maintain the current formatting of the tables.

**References**

1.	Fentress M, Ugarte-Gil C, Cervantes M, et al. Lung Ultrasound Findings Compared with Chest X-Ray Findings in Known Pulmonary Tuberculosis Patients: A Cross-Sectional Study in Lima, Peru. *Am J Trop Med Hyg*. 2020;103(5):1827-1833. doi:10.4269/AJTMH.20-0542

2.	Fentress M, Henwood PC, Maharaj P, et al. Thoracic ultrasound for TB diagnosis in adults and children. *Public Heal Action*. 2022;12(1):3-6. doi:10.5588/pha.21.0072

3.	Correa M, Zimic M, Barrientos F, et al. Automatic classification of pediatric pneumonia based on lung ultrasound pattern recognition. *PLoS One*. 2018;13(12):e0206410. doi:10.1371/journal.pone.0206410

4.	Van Hoving DJ, Kenge AP, Maartens G, Meintjes G. Point-of-care ultrasound predictors for the diagnosis of tuberculosis in HIV-positive patients presenting to an emergency center. *J Acquir Immune Defic Syndr*. 2020;83(4):415-423. doi:10.1097/QAI.0000000000002279

5.	Griesel R, Cohen K, Mendelson M, Maartens G. Abdominal ultrasound for the diagnosis of tuberculosis among human immunodeficiency virus-positive inpatients with world health organization danger signs. *Open Forum Infect Dis*. 2019;6(4):1-7. doi:10.1093/ofid/ofz094

6.	Van Hoving DJ, Griesel R, Meintjes G, Takwoingi Y, Maartens G, Ochodo EA. Abdominal ultrasound for diagnosing abdominal tuberculosis or disseminated tuberculosis with abdominal involvement in HIV-positive individuals. *Cochrane Database Syst Rev*. 2019;2019(9). doi:10.1002/14651858.CD012777.pub2

7.	Bobbio F, Di Gennaro F, Marotta C, et al. Focused ultrasound to diagnose HIV-Associated tuberculosis (FASH) in the extremely resource-limited setting of South Sudan: A cross-sectional study. *BMJ Open*. 2019;9(4):1-7. doi:10.1136/bmjopen-2018-027179
